# PP13 Cost-Effectiveness Analysis Of Antiandrogen Therapies For Metastatic Castration-Sensitive Prostate Cancer: The Brazilian Healthcare System Perspective

**DOI:** 10.1017/S0266462324001867

**Published:** 2025-01-07

**Authors:** Ludmila Gargano, Layssa Oliveira, Haliton Alves De Oliveira, Rosa Lucchetta

## Abstract

**Introduction:**

Brazilian Unified Health System (SUS) Clinical Practice Guidelines (CPG) for prostate cancer recommend the use of androgen deprivation therapy (ADT) ± docetaxel for metastatic castration-sensitive patients (mCSPC). However, new medications have been developed since the last CPG update. We assessed the cost-effectiveness of abiraterone, apalutamide, darolutamide, and enzalutamide for mCSPC as part of the CPG update process.

**Methods:**

A cost–utility analysis was built, with partitioned survival model composed of three states: progression-free, evidence of radiological progression, and death. The overall survival (OS) and progression-free survival (PFS) curves from docetaxel STAMPEDE trial were extrapolated using different distributions for a 20-year horizon. Visual inspection, clinical plausibility, and Akaike information criterion (AIC)/Bayesian information criterion (BIC) tests were considered to choose the best fit, which were adjusted by hazard ratios (HR) from indirect comparisons of interventions with docetaxel. Utility and disutility values were identified from the literature. Direct costs of medications and pre- and post-progression disease monitoring were considered. We considered a BRL120,000/quality-adjusted life year (QALY) (i.e., USD24,511) cost-effectiveness threshold.

**Results:**

When compared to docetaxel+ADT, interventions with higher effectiveness gains were abiraterone+docetaxel and darolutamide+docetaxel (0.94 and 0.99 QALY, respectively). Apalutamide and enzalutamide showed the highest incremental cost (BLR637,342 and BLR516,313, [i.e., USD130,187 and USD105,465], respectively). Abiraterone monotherapy or +docetaxel presented the lowest incremental cost–utility ratio (ICUR) (BLR84,960 and BLR79,428/QALY gained [i.e., USD17,354 and USD16,224], respectively) and may be cost effective for the SUS. Apalutamide, enzalutamide, and darolutamide may not be cost effective for the SUS as ICUR were higher than the cost-effectiveness threshold. Probabilistic sensitivity analyses or using different distributions for survival curves confirmed the direction of the findings.

**Conclusions:**

Recent studies have demonstrated that adding docetaxel or antiandrogen drugs to ADT in men with mCSPC can improve OS and PFS compared with ADT alone. However, in this analysis, only abiraterone was cost effective, and the incorporation of apalutamide, darolutamide, and enzalutamide would require substantial price reductions to be cost effective for the SUS.

